# Improving disseminated histoplasmosis diagnosis in HIV/AIDS patients in Suriname: The role of a urine lateral flow assay

**DOI:** 10.1371/journal.pntd.0012272

**Published:** 2024-06-28

**Authors:** Lycke Woittiez, Stefan Vestjens, Terrence Mawie, Ed IJzerman, Pieter-Jan Haas, Ferry Hagen, Jimmy Roosblad, Stije Leopold, Maaike D. van Schagen, Michèle van Vugt, Stephen Vreden

**Affiliations:** 1 Department of Internal Medicine, Academic Hospital Paramaribo, Paramaribo, Suriname; 2 Department of Internal Medicine, Amsterdam University Medical Center, Amsterdam, The Netherlands; 3 Department of Medical Microbiology and Immunology, Diakonessenhuis, Utrecht, The Netherlands; 4 Department of Medical Microbiology, Academic Hospital Paramaribo, Paramaribo, Suriname; 5 Department of Medical Microbiology, University Medical Center Utrecht, Utrecht, The Netherlands; 6 Westerdijk Fungal Biodiversity Institute, Utrecht, The Netherlands; 7 Department of Clinical Chemistry, Academic Hospital Paramaribo, Paramaribo, Suriname; 8 Faculty of Medicine, Vrije Universiteit Amsterdam, Amsterdam, The Netherlands; 9 Foundation for the advancement of Scientific Research in Suriname, Paramaribo, Suriname; OSWALDO CRUZ FOUNDATION, BRAZIL

## Abstract

Histoplasmosis is a frequent cause of infections in people living with HIV/AIDS (PLWHA). This study introduces the application of a *Histoplasma capsulatum* urine antigen lateral flow assay (LFA) for diagnosing disseminated histoplasmosis in PLWHA in Suriname. The LFA’s diagnostic accuracy was compared with the current diagnostic approach, aiming to assess whether this test resulted in improved early detection and management. Additionally, the prevalence of histoplasmosis among advanced stage HIV patients without clinical suspicion of infection was evaluated using the same LFA. In total, 98 patients were included in the study, of which 58 were classified as “possible disseminated histoplasmosis (DH)” based on clinical criteria and 40 as “controls”. Of these possible DH cases, only 19 (32.7%) had a positive LFA. During the study, decisions for treatment were made without the treating physician being aware of the LFA result. Only 55% of the patients who started treatment for histoplasmosis based on clinical criteria had a positive LFA, and 21% of untreated patients had a positive LFA. This study shows that combining clinical signs with LFA results enhances diagnostic accuracy and is cost effective, resulting in better treatment decisions.

## Author summary

People living with HIV in Suriname often present to care in a late stage of their infection. A common infection in these patients is Disseminated Histoplasmosis (DH). This is caused by a fungus, *Histoplasma capsulatum*. DH is currently difficult to diagnose. This study evaluated the use of a urine test for the diagnosis of DH, called LFA, in patients with advanced-stage HIV-infection.

In the study, 98 advanced-stage HIV infected patients were included. Of these, 58 had signs and symptoms that could be attributable to DH. The other 40 patients were not suspected of DH. In all these patients the usual workup was done. The urine histoplasmosis LFA was carried out afterwards, so the result did not influence clinical decisions. We compared the clinical diagnoses of all patients to the results of the LFA.

The results show that only 55% of the patients with a clinical diagnosis of DH and who started antifungal treatment had a positive LFA. Of the untreated patients 21% had a positive LFA. We conclude that treatment decisions solely based on clinical signs and symptoms lead to both over- and undertreatment and that adding this test to the clinical workup of patients suspected of DH, is cost-effective.

## Introduction

Infection by *Histoplasma capsulatum* can result in various clinical presentations including acute or chronic pulmonary disease, mediastinal disease and disseminated infection [1]. Although histoplasmosis outbreaks are often associated with contact with bird or bat droppings, in sporadic cases patients recall these exposures in only 25% of cases [2]. Disseminated histoplasmosis (DH) is a severe infection with a high mortality, most often observed in advanced HIV patients with CD4 counts <150 cells/μl [1]. Suriname is a low middle income country (LMIC) in South America with a multi-ethnic population and an estimated HIV prevalence of 1.6% [3]. First presentation of HIV infection is often late, not rarely in the stage of clinical AIDS. In Suriname, histoplasmosis is one of the most important co-infections in patients with AIDS. The exact incidence of histoplasmosis in Suriname is unknown, because no previous studies have been performed. A study performed in neighboring French Guiana in 2020 showed that DH was the most common opportunistic infection in patients with HIV [4], and another study estimated the annual histoplasmosis incidence per 100 PLWH at 2.36 [5]. Prevalence of histoplasmosis in the general population was investigated in Suriname in 1953, using a histoplasmine skin test. This test was positive in 43.1% of investigated subjects [6]. Therefore, we suspect that histoplasmosis is very common in Suriname.

Histoplasmosis can be diagnosed by a variety of diagnostic modalities. The golden standard for diagnosis is either culture or PCR. However, culture growth of histoplasmosis is slow and sensitivity of cultures is low [7–9]. PCR on normally sterile tissues has a high specificity and sensitivity [8,9]. In LMIC’s such as Suriname, PCR equipment and consumables are not routinely available, and no biosafety level three facility is present for culture determination. Histological examination of biopsies of affected organs such as bone marrow, liver or skin has relatively high sensitivity but is invasive and time-consuming and therefore not routinely performed [7,10]. DH can also be confirmed by serum buffy coat light microscopy, though this method has a sensitivity of less than 30% [11]. Consequently, DH diagnosis in our setting is mainly based on clinical symptoms, leading to potential under- and overdiagnosis and inappropriate treatment, affecting patient outcomes and healthcare costs [1,12,13].

An alternative to the above-described diagnostic methods is a *Histoplasma* antigen test [7,14]. Recent studies evaluating the use of a MiraVista *Histoplasma capsulatum* antigen lateral flow assay (LFA) for the diagnosis of disseminated histoplasmosis in PLWHA have shown a sensitivity between 79–96% and a specificity between 90–99% [15–17]. It has been shown that in areas where *Histoplasma* rapid antigen tests were implemented, the diagnostic rate of DH increased significantly. Since this allowed for earlier initiation of therapy the mortality of DH decreased [13,18].

In this study, we assessed the value of the MiraVista *Histoplasma capsulatum* antigen LFA, comparing its results with our current diagnostic approach in HIV patients with suspected DH. Additionally, we evaluated the prevalence of positive LFA results among advanced stage HIV patients without a clinical suspicion of DH.

## Materials and methods

Ethical approval for this study was obtained from the National Research Ethics committee of Suriname (letter number 020/23).

This single center prospective cohort study was conducted at the Academic Hospital Paramaribo, Suriname. HIV patients with CD4 counts of <200 cells/μl or a WHO clinical stage three or four for HIV/AIDS were included [19]. CD4 counts were unavailable during a large part of the study period due to a nationwide stockout of reagents. We classified patients as “possible DH” cases when at least three of the following symptoms were present: fever, pancytopenia (with low values in at least two out of three blood lines), weight loss, skin or mucosal lesions, pulmonary infiltrates demonstrated by chest X-ray, lymphadenopathy or hepato- and/or splenomegaly. These signs and symptoms were selected based on previous articles on DH [20,21]. Patients who had already received treatment for histoplasmosis more than four weeks before enrollment were excluded.

A patient who was defined as a possible case, was not automatically treated for histoplasmosis. The attending physician determined the most likely diagnosis based on clinical symptoms and available laboratory tests and then chose at his discretion, the most appropriate treatment. In severe DH, conventional amphotericin was started, followed by itraconazole once the patient was clinically stable. The itraconazole was continued for six months. Controls consisted of patients with low CD4 counts or a WHO clinical stage three or four who were admitted to the hospital or visited the outpatient clinic, but without suspicion of DH.

Written informed consent was obtained from all patients. A questionnaire was conducted in which current symptoms, social conditions and questions about the HIV infection were included. Patient characteristics, laboratory values at the time of inclusion, clinical information about the HIV infection, and adherence to antiretroviral therapy were registered. Patients were also asked about exposure to bird or bat droppings. Additionally, the clinical diagnosis at admission and discharge, treatment regimens, and the outcome of hospitalization were documented for all cases.

In both suspected cases and controls, the standard diagnostic workup for patients with HIV with a low CD4 count in Suriname was performed. This included laboratory investigation, chest X-ray, blood cultures, CD4 counts (when available) and HIV viral load. Serum buffy coat microscopy for presence of *H*. *capsulatum* yeast cells was carried out exclusively in the cases.

Urine was collected from both cases and controls. The collected urine (≥ five ml) was stored at -20°C, for later batch testing, for which the LFA from MiraVista Diagnostic Laboratories (Indianapolis, IN, USA) was deployed. This assay is a qualitative lateral flow-based immunoassay. It employs polyclonal antibodies in the direct detection of Histoplasma antigen in urine. Urine specimens were tested according to the manufacturer’s instructions. The presence of test lines was independently and visually assessed by two individuals.

It is important to note that the results of the urine antigen tests did not influence clinical decision making due to the batch nature of the testing. However, patients who were retrospectively found to have a positive *Histoplasma* urine antigen test and had not received antifungal treatment were invited for an additional outpatient clinic visit for a reevaluation for active histoplasmosis. Treatment for histoplasmosis was only offered when signs of active disease were identified during this reevaluation. Follow-up was continued until eight months after inclusion.

For statistical analysis SPSS version 25 (IBM, Armond, NY, USA) was used. We distinguished between possible cases and controls and compared treatment and mortality rates between both groups using frequencies and percentages.

## Results

**Fig 1:**
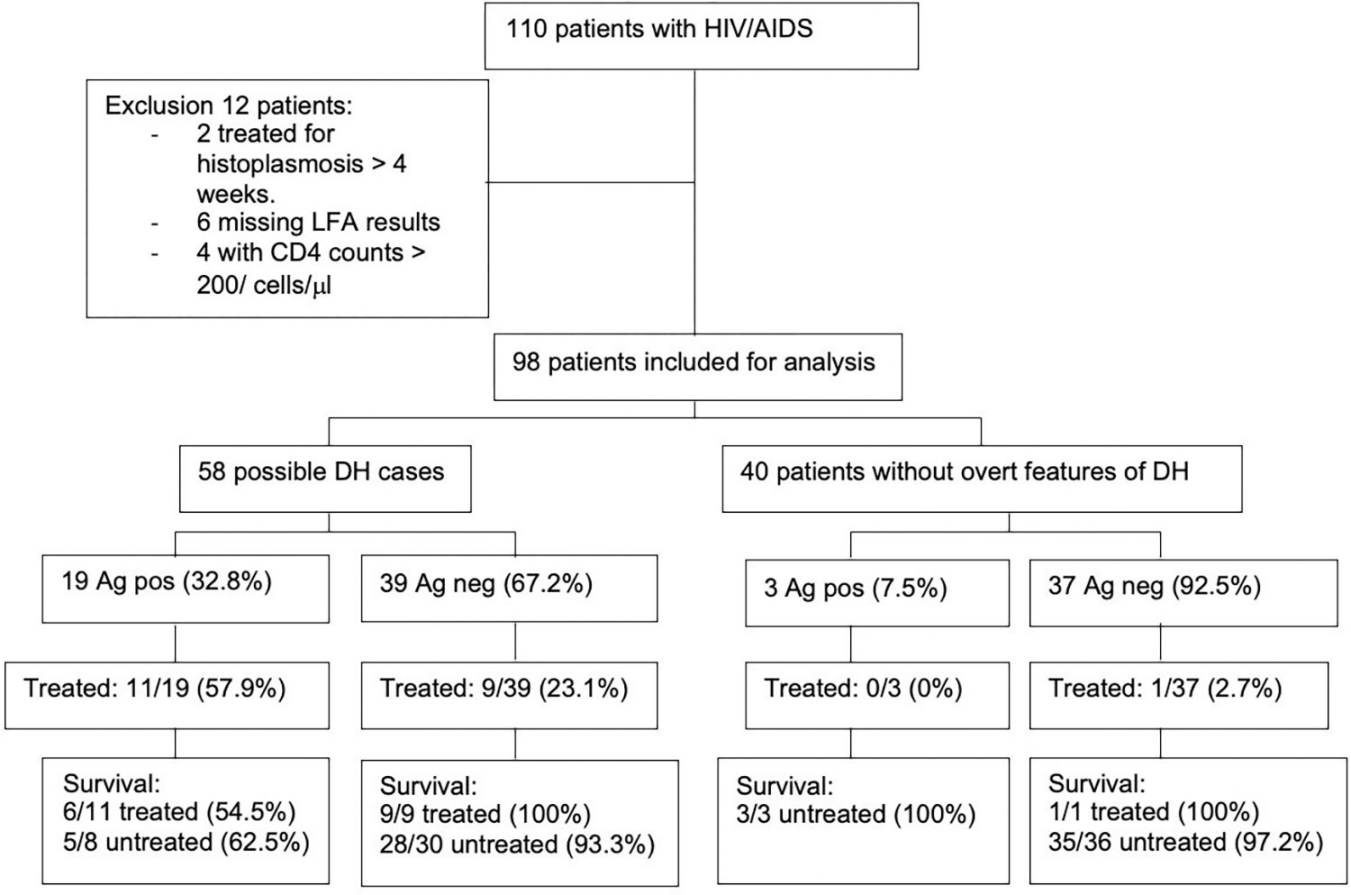
Flowchart of patient population. DH: disseminated histoplasmosis.

Between January 26, 2022 and December 8, 2022, a total of 110 patients were initially enrolled. Twelve patients were excluded from the analyses for various reasons (Fig 1) resulting in a sample of 98 patients.

Of the 98 included patients in the study, 58 (59.2%) met the criteria for possible DH, while 40 (40.8%) served as negative controls. Table 1 presents the baseline characteristics of cases and controls. Notably, the LFA was positive in 19 cases (32.8%) and in three controls (7.5%). All 5 patients with a positive buffy coat had a positive LFA test as well.

Details of the 22 patients with a positive LFA are presented in Table 2.

**Table 1:** Baseline characteristics of cases and controls based on clinical criteria.

	Possible DH cases (n = 58)	Patients without overt features of DH (n = 40)	Missing
Sex (m)	38 (65.5%)	19 (47.5%)	
Age (mean and SD)	40.05 (12.73)	42.25 (13.25)	0
Contact with bat or bird droppings	18 (36.0%)	8 (21.6%)	11
Antigen positive	19 (32.8%)	3 (7.5%)	0
Buffy coat positive (n tested = 84)	5 (9.8%)	0 (0%)	14
**Immune status**			
Latest CD4 count (mean + SD)	88.0 (52.3)	68.6 (25.65)	79
Lowest CD4 count (mean + SD)	128.4 (121.1)	128.1 (164.5)	28
Never on ART	26 (49.1%)	14 (38.9%)	9
Stopped ART	24 (45.3%)	21 (58.3%)	9

ART = Antiretroviral therapy. DH = Disseminated Histoplasmosis

**Table 2:** Characteristics of the patients with a positive LFA.

Number	Case / control	Treated	Outcome at eight months	Clinical features
**A008**	Case	Yes	Alive	Recurrent fever and pancytopenia. Itraconazole and antiretroviral therapy repeatedly stopped and started during and between admissions. Doing well after eight months.
**A017**	Case	Yes	Alive	During admission treated with itraconazole and started ART. Doing well after eight months.
**A046**	Case	Yes	Alive	Pancytopenia and cough. Started itraconazole and ART. Doing well after eight months.
**A056**	Case	Yes	Alive	Lymphadenopathy. Treated with itraconazole and ART. Doing well after eight months.
**A062**	Case	Yes	Alive	New HIV diagnosis, pneumonia. Started itraconazole and ART. Doing well after eight months.
**A108**	Case	Yes	Alive	COVID, pancytopenia, elevate liver enzymes. Started itraconazole and ART. Doing well after eight months.
**A001**	Case	Yes	Died	Histoplasmosis, buffy coat microscopy confirmed. Treatment started but refused by patient.
**A002**	Case	Yes	Died	COVID infection, suspected histoplasmosis, treated with itraconazole.
**A007**	Case	Yes	Died	Candida esophagitis and histoplasmosis. Started itraconazole and ART. Died shortly after discharge.
**A013**	Case	Yes	Died	Attempted suicide with chloride, respiratory failure. Started itraconazole and ART. Recovered during admission but died shortly after discharge. Cause of death unknown.
**A025**	Case	Yes	Died	Respiratory failure. Died soon after admission.
**A047**	Case	No	Alive	Known HIV infection, lost to follow up. Presented with weakness, fever, shortness of breath, weight loss, abdominal pain. Started itraconazole and ART two months after initial presentation. Doing well after eight months.
**A061**	Case	No	Alive	Known HIV infection, lost to follow up. Presented with weight loss, fever and cough. After restarting ART clinical improvement, but one month later again lost to follow up. Eight months after admission the patient is alive.
**A065**	Case	No	Alive	New HIV diagnosis with weight loss, diarrhea, fever, severe oral candidiasis and abdominal lymphadenopathy. ART and fluconazole were started. Fluconazole was continued for five months. Eight months after presentation the patient was alive and well.
**A092**	Case	No	Alive	Admitted with gastro-enteritis. New HIV diagnosis, CD4 26 cells/μl. Started with ART. Eight months later alive and well.
**A099**	Case	No	Alive	Admitted with fever, weight loss and weakness. cryptococcal Ag was positive. Treatment was started with amphotericin B, followed by fluconazole 800mg and later fluconazole 150mg. During follow up eight months later patient is alive and well.
**A082**	Case	No	Died	Admitted with HIV and severe wasting. During admission ART was started, however after discharge all treatment was stopped by the patient, and she died.
**A109**	Case	No	Died	Admitted with fever and respiratory symptoms. Died in the ICU <24hrs after admission.
**A029**	Case	No	LTFU	Admitted with COVID-19. Elevated liver enzymes and lymphadenopathy. After discharge lost to follow up.
**Negative controls**				
**A035**	Control	No	Alive	Presented with weight loss and a new HIV diagnosis. No other symptoms. Antiretroviral therapy was started. After eight months nine kilogram of weight gain, no clinical symptoms.
**A057**	Control	No	Alive	Known HIV infection, lost to follow up. Presented with diarrhea, abdominal pains, weight loss. Started ART and fluconazole prophylaxis. Eight months later alive and well.
**A097**	Control	No	Alive	Admitted with a new HIV diagnosis, CD4 112 cells/μl. Acute gastro-enteritis and an oral *Candida* infection. Received fluconazole 150mg for one week and started ART. Eight months later alive and well.

ART = antiretroviral therapy

In the 58 patients defined as possible DH cases, histoplasmosis treatment was started in 19 patients. Only 11 of those patients (55%) had a positive urine antigen LFA. In retrospect, in the other eight patients an alternative diagnosis could be made (Table 3).

**Table 3:** Possible alternative diagnosis in nine possible DH cases with negative histoplasmosis LFA who received treatment.

	Clinical features / alternative diagnosis
**A004**	Suspected *Pneumocystis* pneumonia.
**A006**	Suspected *Pneumocystis* pneumonia.
**A018**	CMV and HBV infection.
**A019**	Fever, cachexia. Possible urosepsis (cultures blood and urine with *Klebsiella pneumoniae*) or *Pneumocystis* pneumonia.
**A022**	Pneumonia (causative pathogen unknown).
**A037**	Pneumococcal empyema.
**A044**	*Staphylococcus aureus* bacteremia.
**A073**	Gastro-enteritis, lymphadenopathy. No other infectious focus found.
**A088**	Confusion, CT scan no abnormalities. Drugs abuser. Left the hospital during evaluation.

CMV = Cytomegalovirus. HBV = Hepatitis B virus

Thirty-eight patients in the group of possible DH were not treated based on the decision of the treating physician. In this group, eight (21%) were later tested LFA positive. Details of these eight cases are presented in Table 2. Two patients died; one patient was lost to follow up. The remaining five patients recovered and were doing well at eight months after inclusion. Two had been treated with high doses of fluconazole for severe *Candida* esophagitis or a positive cryptococcal antigen test. The other three patients recovered without any antifungal medication.

The three controls with a positive LFA were re-evaluated and since even then active histoplasmosis was not likely, they were not treated; all of these three patients remained well.

Only 6 (27.3%) of the patients with a positive LFA reported exposure to bat or bird droppings.

Of the possible DH cases with a positive LFA, five of the eleven (45.5%) who received treatment and two of the eight (25%) who did not, died during admission or shortly after discharge (Table 2).

## Discussion

The primary goal of this study was to determine whether the use of the MiraVista *Histoplasma* capsulatum urine antigen LFA would improve the diagnosis of patients with DH in Suriname, compared to our current approach where suspicion of DH and start of treatment is based on clinical symptoms. Assessment of a direct effect of the LFA on treatment decisions was not included in this study.

The first step in our diagnostic process was to distinguish between cases and controls, using a combination of clinical signs and symptoms. This grouping proved to be adequate in ruling out DH, but not in diagnosing DH. In the group of possible DH cases, a clinical decision was made whether or not to start treatment. In retrospect, in the group of possible DH cases in which treatment was started, only 55% had a positive LFA. Also, eight cases (21%), who later proved to have a positive LFA were not treated, of which two deceased (25%). Of the cases with a positive LFA who were treated, 45.5% died. The high mortality in both the treated and untreated patients underlines the reported severity of DH [13,22,23].

Our data show that clinical criteria alone are insufficient in determining whether a patient with late-stage HIV infection is likely to have DH in our setting and if treatment should be started. This finding is consistent with earlier studies; diagnosing DH by signs and symptoms alone is challenging [13]. Similarly, lack of exposure to risk factors cannot be used to rule out histoplasmosis. In our study, only 27% of patients remembered such exposure, which is consistent with earlier research [2]. The finding that all five cases with a positive buffy coat had indeed a positive LFA, supports the sensitivity of the latter test, but because of the known low sensitivity of microscopic blood diagnosis, it is insufficient to be used as the only determinant of DH [11].

Five of the patients with a positive LFA who recovered without treatment had been defined as possible DH cases. Two of them were treated with high doses of fluconazole for other diagnoses. Although fluconazole is less effective than itraconazole for treating histoplasmosis, it can be effective (especially in high doses), and these patients might therefore also have been successfully treated for histoplasmosis [24]. One patient started itraconazole after the LFA result, two months after initial presentation. The other two patients recovered without any treatment. Three patients in the control group had a positive LFA and survived without treatment. Several explanations can be considered about the patients with a positive LFA who recovered without treatment for histoplasmosis.

The first explanation might be a false positive histoplasmosis LFA result. Cross-reactions are observed mainly in samples from patients with proven diagnosis of paracoccidioidomycosis, blastomycosis and coccidioidomycosis, but cross-reactivity with other mycoses such as *Talaromyces marneffei* and *Aspergillus* species has also been reported [15,17,25,26]. The prevalence of these mycoses in Suriname is unknown. However, they can also be treated with itraconazole [27].

Another explanation could be that the untreated patients did not have disseminated histoplasmosis but a localized infection such as pulmonary histoplasmosis [17]. According to guidelines, treatment for pulmonary and mediastinal histoplasmosis is not necessary in people with an adequate immune system [28]. It is possible that the untreated patients in our cohort had more localized disease, or a relatively good immune system.

A third explanation could be that some patients had a low level of antigenuria. The LFA is a qualitative test that does not discern the level of antigenuria. Elsewhere it has been reported that the level of antigenuria correlates with the severity of disease [14]. It should be considered that the level of fungemia is another important factor in disease progression.

In theory, there could also be consumption of food containing *Histoplasma* antigen, resulting in a false positive LFA in the person eating the food. This has been described in only 1 study [29].

Furthermore, an important part of the treatment of disseminated histoplasmosis is probably the restoration of immunity by starting antiretroviral therapy. Earlier it was shown that non-adherence to ART was an independent risk factor associated with therapy failure, relapse and death [21,28,30,31].

A definite conclusion about the reasons why some patients with a positive LFA recovered without treatment cannot be drawn from our findings.

Nine possible DH cases with a negative LFA had received treatment. On reevaluation, an alternative diagnosis was considered likely. This finding may point to overtreatment due to the inadequacy of decision making by clinical evaluation alone (Table 3).

A second objective of our study was to assess the prevalence of histoplasmosis infections in advanced stage HIV-patients, without suspicion of DH. The LFA was positive in three controls (7.5%). None of these control patients became ill nor developed Immune Reconstitution Inflammatory Syndrome (IRIS) after starting antiretroviral therapy. Unmasking DH IRIS is rare but has been described in case reports [32]. On additional evaluation of these three control patients after the results of the LFA became available, no signs of DH were found. Therefore, we conclude that if no clinical signs of DH are present, a LFA should not routinely be performed in PLWHA in our setting. This approach is different from the advice for cryptococcosis, where patients with a low CD4 count are advised to be screened for the presence of cryptococcal antigen, to allow antifungal treatment in order to prevent IRIS after starting ARV [33].

Antigen detection tests for diagnosis of histoplasmosis were implemented in other countries in the region as well. Studies show a clear increase in diagnostic rate of histoplasmosis after implementation of antigen detection tests [23,34]. The positivity rate varies between 20% to 72.3% [23,35].

An important aspect when considering implementing a new diagnostic test are its costs. This is even more important in LMIC like Suriname. Performing the MiraVista *Histoplasma* LFA is approximately 50 times less expensive than treating DH during six months. It Is therefore easy to conclude that rapid diagnosis of histoplasmosis using a *Histoplasma* LFA in suspected patients is cost-effective.

Limitations of this study include the small number of patients and the limited number of CD4 cell counts that were available during the study period. Furthermore, there is a lack of a good reference tests for DH such as culture, as well as for alternative diagnoses such as *Pneumocystis* pneumonia.

## Conclusions

This study highlights the utility of the *Histoplasma* urine antigen LFA in diagnosing disseminated histoplasmosis in HIV patients in Suriname.

It shows that neither the *Histoplasma* LFA nor the clinical picture alone are sensitive enough to determine which patients should be treated for disseminated histoplasmosis. Combining clinical signs with LFA results enhances diagnostic accuracy and is cost effective, leading to better treatment decisions. Considering the cost-effectiveness of rapid histoplasmosis diagnosis, implementing LFAs in diagnostic protocols could significantly impact patient outcomes and healthcare costs.

The *Histoplasma* urine antigen LFA is a valuable addition to histoplasmosis diagnostics, aiding in accurate and timely diagnosis.

## Supporting information

S1 Table Raw data of patient characteristics and LFA results. (XLSX)
